# The current status of clinical trials on cancer and age disparities among the most common cancer trial participants

**DOI:** 10.1186/s12885-023-11690-9

**Published:** 2024-01-02

**Authors:** Shuang Zhao, Miao Miao, Qingqing Wang, Haijuan Zhao, Han Yang, Xin Wang

**Affiliations:** Clinical Trial Research Center, Beijing Hospital, National Center of Gerontology, Institute of Geriatric Medicine, Chinese Academy of Medical Sciences, Beijing, P.R. China

**Keywords:** Current status, Clinical trial, Age disparities

## Abstract

**Objective:**

To illustrate the status of all cancer clinical trials and characterize clinical trial enrollment disparities in the most common cancer.

**Methods:**

Clinical trial data were extracted from ClinicalTrials.gov website. All searched clinical trials were included in the current status analysis of clinical trials on cancer. Among all the clinical trials, only trials addressing single disease sites of breast, prostate, colorectal, or lung (BPCRL) cancer were included in the age disparities analysis. The difference in median age (DMA) between the trial participant median age and the population-based disease-site-specific median age was calculated for each trial.

**Results:**

A total of 7747 clinical trials were included in the current status analysis of clinical trials on cancer. The number of registered trials had been increasing from 2008 to 2021 (AAPC = 50.60, 95% CI 36.60, 66.00, *P* < 0.05). Of the 7747 trials, 1.50% (116) of the studies were clinical trials for the elderly aged 60 years or older. 322 trials were included in the age disparities analysis. For all trials, the median DMA was − 8.15 years (*P*_25_, *P*_75_, − 10.83 to − 2.98 years, *P* < 0.001). The median DMA were − 9.55 years (*P*_25_, *P*_75_, − 11.63 to − 7.11 years), − 7.10 years (*P*_25_, *P*_75_, − 9.80 to − 5.70 years), − 9.75 years (*P*_25_, *P*_75_, − 11.93 to − 7.35 years), 3.50 years (*P*_25_, *P*_75_, 0.60 to 4.55 years), respectively, for breast cancer, colorectal cancer, lung cancer and prostate cancer.

**Conclusion:**

The numbers of registered clinical trials show an upward trend. Age disparities between trial participants and diagnosed disease population are present in BPCRL cancer trials and appear to be increasing over time. Equitable participation in clinical trials on the basis of age is crucial for advancing medical knowledge and evaluating the safety and efficacy of new treatments that are generalizable to aging populations.

## Introduction

Population aging has substantially contributed to the increasing number of new cancer cases worldwide. In 2018, assessment of global cancer burden showed that 2.3 million new cases occurred in adults aged 80 or older worldwide (13% of all cancer cases). Projections suggest that by 2050, an estimated 6.9 million new cancers will be diagnosed in this age group (20.5% of all cancer cases) [1]. The elderly are the fastest-growing segments of the world's population. Despite shouldering a disproportionate burden of disease and consumption of prescription drugs, the elderly are vastly underrepresented in clinical trials. In particular, older adults over the age of 75 are chronically underrepresented in cancer clinical trials [2]. Ensuring adequate population representation in clinical trials is critical to generate sufficient data on the safety and efficacy of interventions in all age groups. Unfortunately, many drug trials do not include older adults because of concerns about the safety and efficacy of drugs in the older population [3–5]. Randomised controlled trials (RCTs) often establish standard clinical practices. The applicability of the trial results could be compromised by the under-representation of elderly patients [6–8].

The lack of diverse participants in trials is an ethical and scientific issue, because it could limit the application of future therapies. Increasing representation of diverse participants into clinical trials is essential for assessing drug effectiveness and safety. Clinical trials that do not adequately represent the diversity of the population, particularly those most affected by certain diseases, may lead to the results not being generalizable.

Accordingly, it is necessary to analyse the current state of cancer clinical trials and explore the gap between the age of enrollment in clinical trials and the true median age of cancer patients at diagnosis. A study examined age disparities among modern oncologic clinical trials for breast, prostate, colorectal, and lung cancer (the 4 most common disease sites), characterizing the differences between trial participants and the population by disease site. In this analysis, 302 randomised clinical trials before 2017 were included. And this study found trial participants were significantly younger than the population by disease site [9]. Further research is needed to determine whether the age gap improves over time. Therefore, in this study, we first illustrated the status of all clinical trials on cancer and then characterized the clinical trial enrollment disparities in the most common cancer, focusing on improving enrollment to be more representative of the trial population.

## Materials and methods

### Data acquisition and processing

In our study, we used the ClinicalTrials.gov dataset, which is one of the most comprehensive clinical research databases globally. The ClinicalTrials.gov database was frequently selected by other studies to characterize study populations and trends in clinical care and research [10, 11].

**Fig. 1:**
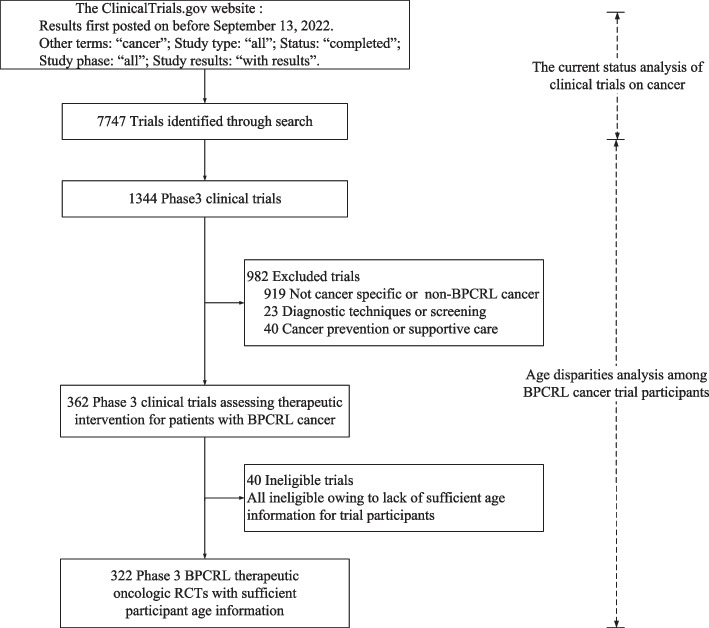
Flowchart of clinical trial screening, eligibility, and inclusion

The trial status in ClinicalTrials.gov registry included active, not recruiting, by invitation, recruiting, suspended, terminated, withdrawn, completed, and unknown. We included completed clinical trials in our analysis. Oncology clinical trials up to September 13, 2022 were searched on the ClinicalTrials.gov website. During the search process, the following advanced search parameters were utilized: other terms: “cancer”; study type: “all studies”; status: “completed”; study phase: “early phase 1, phase 1, phase 2, phase 3, phase 4” and study results: “with results”. A total of 7747 clinical trials were yielded. All 7747 clinical trials were used to analyse the current status of cancer clinical trials. Among all the clinical trials, the phase 3 trials targeting therapeutic intervention were screened to analyse age disparities among cancer trial participants (Fig. 1). Due to all trial-level data were publicly available, informed consent was not obtained.

### Data evaluation

To provide information on the development and status quo of cancer treatment in the clinical setting, study entries were sorted in ascending order according to the date on which the study record first posted on ClinicalTrials.gov. Furthermore, common standardized study parameters were evaluated, such as study type, study phases, ages eligible for study or molecular profile restriction [12]. To provide a more in-depth description for the dataset, we also evaluated the study entries and categorized them according to additional parameters such as recruiting country, sponsoring country, or industry sponsor.

Trials targeting a single disease site of breast, prostate, colorectal or lung cancer were eligible for the analysis of age disparities among participants in the BPCRL cancer trials. Clinical trials that did not provide the median age of participants were not included in the study analysis. To avoid information bias, the two individuals performed clinical trial screening and parameter identification independently. Finally, the two individuals compared decisions and resolved disagreements through discussion.

### Statistical analyses

Statistical analyses were performed at the trial level (each trial is an observation). Trial factors were summarized by frequency and percentage. To analyse the trend of the number of trial registrations over time, we calculated the annual percentage of change (APC) and average annual percent change (AAPC) using the method of joinpoint regression analysis (Joinpoint version 4.9.0.1, February, 2022). Joinpoint regression fits a piecewise linear regression model, which is a special case of linear spline [13]. Early phase 1, phase 1, and phase 2 were combined as early phase, and phases 3 and 4 were combined as advanced phase [14]. The median age of each trial was compared with the median age of the relevant disease site according to the NCI Surveillance, Epidemiology, and End Results (SEER) database [9, 15]. The median age of SEER by disease site was also matched to the trial enrollment time [9]. For each trial, the difference in median age (DMA) was calculated as the trial median age minus the population median age. Independent-samples Mann–Whitney U and Kruskal–Wallis tests were used to compare the DMA of different groups. *P* < 0.05 was set as significant and all *P* values were 2-sided. Analyses were performed using R 3.6.1.

## Results

### The current status analysis of clinical trials on cancer

**Fig. 2:**
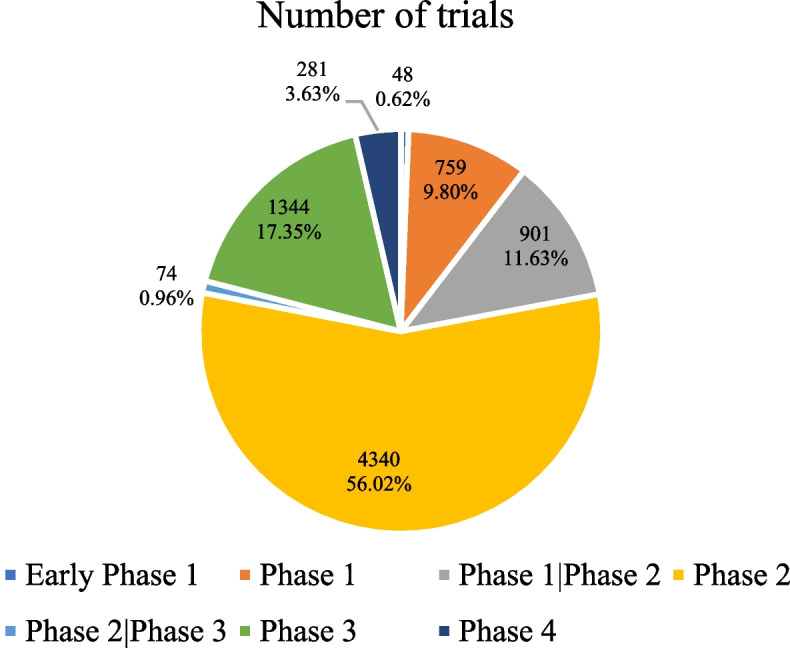
Detailed trial phases of the 7747 clinical trials on cancer

From 2008 to September 2022, a total of 7 747 clinical trials were registered on ClinicalTrials.gov. 1 551 146 participants were enrolled in the 7 747 clinical trials. Of these, only 17 185 participants were 60 years of age or older, and 1.50% (116) of the studies were clinical trials for the elderly aged 60 years or older. Of the 7747 trials designated with a phase, 79.02% were early phase 1, phase 1, and phase 2. The advanced phase included 1625 clinical trials, accounting for 20.98% (Fig. 2).

**Fig. 3:**
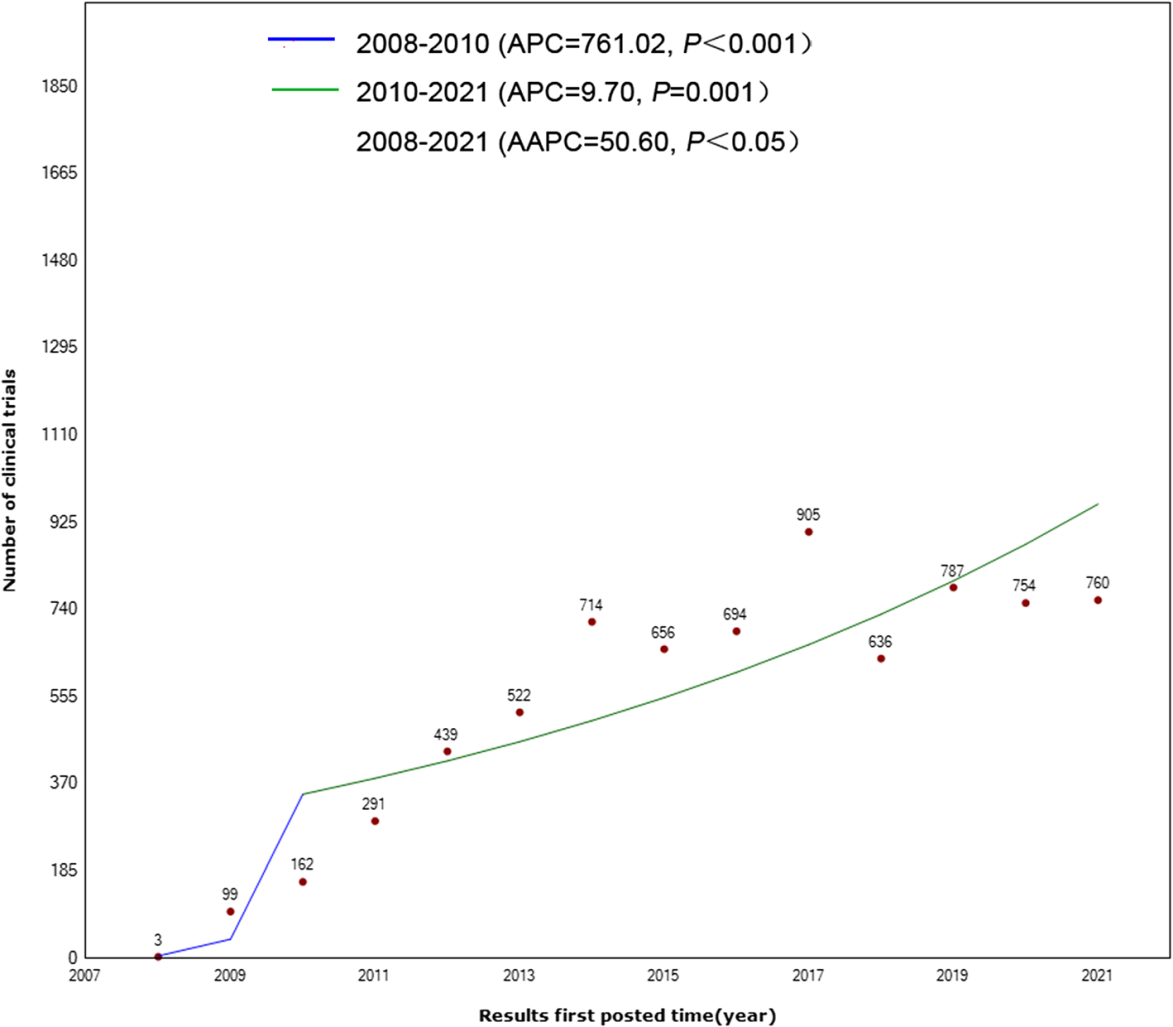
The annual percent change (APC) and average annual percent change (AAPC) of the number of clinical trials during 2008–2021. The solid lines represent the fitted values of the joinpoint regression. The annual percentage change (APC) *P* value corresponds to testing whether the APC is different from zero. Average annual percent change (AAPC) *P* value corresponds to testing whether the AAPC is different from zero

For the trend analysis of the number of trial registrations over time, the APC of the number of trial registrations showed two periods, both of which showed continuous upward trends (2008–2010: APC = 761.02, *P* < 0.001; 2010–2021: APC = 9.70, *P* = 0.001). The number of registered trials had been increasing from 2008 (3 clinical trials) to 2021 (760 clinical trials) (AAPC = 50.60, 95% CI 36.60, 66.00, *P* < 0.05). (Fig. 3).

### Age disparities analysis among BPCRL cancer trial participants

Three hundred twenty-two trials were included in the age disparities analysis (Fig. 1); these trials collectively enrolled a total of 293 267 patients. For all trials, the median DMA was − 8.15 years (*P*_25_, *P*_75_, − 10.83 to − 2.98 years, *P* < 0.001; Table 1). The median DMA were − 9.55 years (*P*_25_, *P*_75_, − 11.63 to − 7.11 years), − 7.10 years (*P*_25_, *P*_75_, − 9.80 to − 5.70 years), − 9.75 years (*P*_25_, *P*_75_, − 11.93 to − 7.35 years), 3.50 years (*P*_25_, *P*_75_, 0.60 to 4.55 years), respectively, for breast cancer, colorectal cancer, lung cancer and prostate cancer.

**Table 1:** Trial factors associated with age disparities

Trial Factor	No. of Trials (%)	Median DMA	DMA (*P*_25_, *P*_75_)	test statistic	*P* Value^*^
All trials	322	-8.15	(-10.83, -2.98)	0.126	< 0.001
Industry funding of trial				-1.375	0.169
Yes	276 (85.71%)	-8.32	(-10.98, -4.55)		
No	46 (14.29%)	-7.00	(-10.08, -0.07)		
International multicenter trial				-1.584	0.113
Yes	230 (74.19%)	-8.90	(-10.93, -4.73)		
No	80 (25.81%)	-6.65	(-11.01, 0.73)		
Age restriction enrollment criterion				-2.783	0.005
Yes	42 (13.04%)	-10.37	(-13.70, -4.33)		
No	280 (86.96%)	-7.95	(-10.38, -2.93)		
Molecular profile restriction criterion				-5.634	< 0.001
Yes	171 (53.11%)	-9.30	(-12.00, -6.90)		
No	151 (46.89%)	-6.40	(-10.00, 1.00)		
Disease site				124.429	< 0.001
Breast	114 (35.40%)	-9.55	(-11.63, -7.11)		
Colorectal	43 (13.35%)	-7.10	(-9.80, -5.70)		
Lung	104 (32.30%)	-9.75	(-11.93, -7.35)		
Prostate	61 (18.95%)	3.50	(0.60, 4.55)		
Modality				84.200	< 0.001
Chemotherapy	129 (40.06%)	-9.00	(-10.90, -6.25)		
Hormone therapy	48 (14.92%)	2.90	(0.13, 4.25)		
Radiation therapy	5 (1.55%)	-2.40	(-11.20, -1.00)		
Surgery	4 (1.24%)	-8.30	(-20.55, -1.83)		
Targeted therapy	132 (40.99%)	-9.30	(-11.48, -6.83)		
Vaccine therapy	4 (1.24%)	3.95	(-0.78, 4.55)		
Results First Posted Time				-0.275	0.783
Before 2017(included 2017)	223 (69.25%)	-8.10	(-11.00, -1.50)		
After 2017	99 (30.75%)	-8.20	(-10.30, -4.00)		
Allocation					
Non-Randomized	6 (2.05%)	-9.10	(-14.08, -8.80)	-1.307	0.191
Randomized	287 (97.95%)	-8.30	(-11.00, -4.50)		
Masking				8.096	0.088
None (Open Label)	204 (63.35%)	-7.90	(-11.00, -3.63)		
Single	4 (1.24%)	-10.10	(-13.93, -6.07)		
Double	40 (12.42%)	-9.75	(-11.53, -7.38)		
Triple	27 (8.39%)	-7.20	(-10.00, 0.80)		
Quadruple	47 (14.60%)	-7.30	(-10.40, 0.10)		

Other *P* values represent the statistical difference in DMA between different groups by trial factors

^*^ All trials *P* value corresponds to testing whether the DMA is significantly different from zero

There was no significant age differences between industry-funded trials, with a median DMA of − 8.32 years for industry-sponsored trials compared with − 7.00 years for non-industry-sponsored trials (*P* = 0.169; Table 1). In addition, there was no significant difference for age disparity between international multicenter trials and non-international multicenter trials (*P* = 0.113). Trials with age restriction enrollment criterion (42 of 322 trials; 13.04%) or restricting molecular profile criterion (171 of 322 trials; 53.11%) were associated with larger DMA (Table 1). Among therapy trials, those that conducted with targeted therapy were associated with a larger DMA, followed by chemotherapy and surgery.

Similarly, sensitivity analyses of US-only trials showed a median DMA of -6.35 years (*P*_25_, *P*_75_, − 10.00 to − 0.33 years, *P* = 0.010). The median DMA were − 10.30 years (*P*_25_, *P*_75_, − 12.00 to − 1.50 years), − 6.60 years (*P*_25_, *P*_75_, − 10.10 to − 5.90 years), − 6.70 years (*P*_25_, *P*_75_, − 7.00 to − 5.00 years), 2.00 years (*P*_25_, *P*_75_, -1.65 to 4.60 years), respectively, for breast cancer, colorectal cancer, lung cancer and prostate cancer (Table 2).

**Table 2:** US-only trials factors associated with age disparities

Trial Factor	No. of Trials	Median DMA	*P*_25_, *P*_75_	test statistic	*P* Value^*^
US-only trials	44	-6.35	-10.00,-0.33	1.626	0.010
Industry funding of trial				-0.504	0.614
Yes	30 (68.18%)	-6.20	-8.53,-1.38		
No	14 (31.82%)	-7.00	-10.08,0.43		
Age restriction enrollment criterion				-1.557	0.120
Yes	6 (13.64%)	-9.50	-14.20,-1.75		
No	38 (86.36%)	-6.20	-9.25,0.18		
Molecular profile restriction criterion				-0.396	0.692
Yes	15 (34.09%)	-6.30	-7.00,-2.20		
No	29 (65.91%)	-6.40	-10.10,-0.10		
Disease site				14.909	0.002
Breast	11 (25.00%)	-10.30	-12.00,-1.50		
Colorectal	9 (20.45%)	-6.60	-10.10,-5.90		
Lung	11 (25.00%)	-6.70	-7.00,-5.00		
Prostate	13 (29.55%)	2.00	-1.65,4.60		
Modality				12.481	0.029
Chemotherapy	21 (47.73%)	-9.00	-11.30,-5.35		
Hormone therapy	5 (11.36%)	2.00	-3.00,4.25		
Radiation therapy	1 (2.27%)	/	/		
Surgery	1 (2.27%)	/	/		
Targeted therapy	14 (31.82%)	-6.20	-7.00,-1.67		
Vaccine therapy	2 (4.55%)	1.20	/		
Results First Posted Time				1.177	0.125
Before 2017(included 2017)	30 (68.18%)	-5.90	-7.70,1.25		
After 2017	14 (31.82%)	-8.00	-11.25,-3.20		
Allocation				-0.203	0.839
Non-Randomized	5 (11.36%)	-2.20	-16.30,6.00		
Randomized	39 (88.64%)	-6.40	-10.00,-1.00		
Masking				5.334	0.255
None (Open Label)	25 (56.82%)	-6.30	-8.50,-0.55		
Single	3 (6.82%)	-10.10	/		
Double	6 (13.63%)	-6.85	-10.15,0.43		
Triple	4 (9.09%)	-5.85	-7.68,0.25		
Quadruple	6 (13.64%)	-2.90	-9.75,5.63		

Other *P* values represent the statistical difference in DMA between different groups by trial factors

^*^US-only trials *P* value corresponds to testing whether the DMA is significantly different from zero

## Discussion

Our research illustrated the status of all cancer clinical trials and analysed the enrollment disparities among the most common cancer trial participants. We found an upward trend in the number of registered clinical trials. And the age gap between trial participants and the diagnosed disease population was present in BPCRL cancer trials.

From 2008 to 2022, there was an upward trend in the number of clinical trial registrations, which could be due to an increase in the number of clinical trials conducted or an increasing number of journals, government funding agencies, universities, and hospitals required trials to be registered. Among all clinical trials, relatively few had been conducted in the elderly population only, which was consistent with other reported data result [16]. The Annual Report on the Progress of Clinical Trials for New Drug Registration in China showed that the trend in the number and proportion of clinical trials in the elderly population remained consistent when comparing data from the last three years. Clinical trials conducted only in the elderly population accounted for no more than 0.2% of all clinical trials in all years [16]. This reflected the enrollment disparities in clinical trials.

Enrollment disparities in clinical trials have been recognized for many years. While two-thirds of cancer patients are over 65 years old, only about 25% of cancer trial participants reach that age [17]. Ethan et al. reported on the factors associated with age disparities among cancer clinical trial participants. They found that trial participants were significantly younger than the population by disease site and the age gap was greater for industry-funded trial participants, which was consistent with our findings [9]. We also found that age disparities between trial participants and the diagnosed disease population appeared to be widening following the BPCRL cancer trial reported by Ethan et al. in 2017. This is true not only in the field of cancer, but also in other disease areas, such as cardiovascular diseases (CVDs). CVDs are the leading cause of death globally, causing an estimated 17.9 million deaths each year [18]; and 65% of those diagnosed are over 65 years of age. Despite these statistics, only 42.5% of participants in clinical trials for cardiovascular disease are over the age of 65, and 12.3% are over the age of 75 [19]. The participation of these older populations in clinical trials is also low in research on Alzheimer’s disease, arthritis, epilepsy and many other diseases [20].

The key reasons that the age of clinical trial participants is lower than the age of diagnosis in the population are typically due to a combination of challenges and barriers faced by both sponsors and older adults. These barriers include comorbidities and polypharmacy. Both may affect the attainment of trial safety or efficacy endpoints. Operational challenges include difficulties in recruitment or retention patients, obtaining informed consent, financial constraints, communication issues (e.g., hearing difficulties and visual impairment), and physical inflexibility, which may limit transportation options to clinical sites. This barrier has led to limitations in age-based exclusion criteria and a preference for including younger participant with a low risk of adverse outcomes in clinical trials [21]. In addition, older adults are more likely to experience adverse effects due to changes in pharmacokinetics (PK) and pharmacodynamics (PD), possible comorbidities, and concomitant therapies that may interact with investigational drugs. These adverse effects may be more severe or less tolerate and have more serious consequences compare with younger participants [22].

The inclusion of elderly patients in clinical trials is undoubtedly important. Decentralized clinical trials (DCT) could reduce barriers and facilitate appropriate participation of older participants. By conducting clinical trials remotely, participants could participate in the research from their own comfortable homes. A recent survey reported that 74% of seniors preferred this option to a clinic visit [23]. The Food and Drug Administration (FDA) also issues DCT draft guidance and encourages sponsors to broaden cancer clinical trial eligibility criteria to enhance the generalizability of trial results and develop strategies for recruiting patients that reflect the intended population [24, 25].

This study had several strengths. Our study described the status of cancer clinical trials up to 2022 and analysed the age disparities among BPCRL cancer trial participants, which provides evidence to support the inclusion of more elderly patients in clinical trials. Further, in the data acquisition and evaluation, two individuals independently performed trial screening and parameter identification to avoid the information bias. Meanwhile, our research had several limitations. First, selection bias could arise from not including clinical trials registered in other registries, such as International Standard Randomised Controlled Trial Number (ISRCTN) registry, European Union Drug Regulating Authorities Clinical Trials (EudraCT) Database. Further validations in different clinical trial registries are necessary to increase the strength of medical evidence. Secondly, we only considered completed clinical trials with results, and thus we were not able to analyse whether the age disparities improved in more recently initiated trials. It is required to conduct further studies from a wider range of data sources. Third, the disease sites included in this study represent the most common cancer, and these sites may not be representative of the entire cancer trial. Additionally, the median age of the population by disease site was based on US SEER data. The majority of included trials (230 of 322; 71.43%) were multinational, and 48 trials (14.91%) were enrolled in a country other than the United States. Therefore, our study was limited by the extrapolation of US demographics to other countries. Nevertheless, we performed sensitivity analyses of US-only trials and obtained the same conclusions. Moreover, the median age, as an indicator, provides limited information on the exact proportion of elderly patients in a certain study. The median was chosen because of the heterogeneity in the age distribution reported by each trial and was compared as a common indicator for each trial, which was also consistent with the previous studies [9]. Lastly, SEER captures patients with relevant diagnoses, not just those treated; the median age of SEER may also disproportionately exclude older cancer patients due to a number of possible factors. Consequently, this analysis may underestimate the extent of age differences among trial participants.

## Conclusions

Our study demonstrated future cancer clinical trials need to include a wider range of patients on the basis of age. Equitable participation in clinical trials contributed to advancing medical knowledge and evaluating the safety and efficacy of new treatments that are generalizable to aging populations.

## Data Availability

The data that support the findings of this study are openly available in the ClinicalTrials.gov. Database at https://clinicaltrials.gov/

## Abbreviations

BPCRL: Breast, prostate, colorectal, or lung
DMA: Difference in median age
RCTs: Randomised controlled trials
APC: Annual percentage of change
AAPC: Average annual percent change
SEER: Surveillance, Epidemiology, and End Results
CVDs: Cardiovascular diseases
PK: Pharmacokinetics
PD: Pharmacodynamics
DCT: Decentralized clinical trials
FDA: Food and Drug Administration
ISRCTN: International Standard Randomised Controlled Trial Number
EudraCT: European Union Drug Regulating Authorities Clinical Trials

## References

[1] Pilleron S, Soto-Perez-de-Celis E, Vignat J (2021). Estimated global cancer incidence in the oldest adults in 2018 and projections to 2050. Int J Cancer.

[2] Singh H, Kanapuru B, Smith C (2017). FDA analysis of enrollment of older adults in clinical trials for cancer drug registration: A 10-year experience by the U.S. Food and Drug Administration. JCO..

[3] Langford AT, Resnicow K, Dimond EP (2014). Racial/ethnic differences in clinical trial enrollment, refusal rates, ineligibility, and reasons for decline among patients at sites in the National Cancer Institute's community cancer centers program. Cancer.

[4] Hurria A, Levit LA, Dale W (2015). Improving the evidence base for treating older adults with cancer: American Society of Clinical Oncology statement. J Clin Oncol.

[5] Giovanazzi-Bannon S, Rademaker A, Lai G, Benson AB (1994). Treatment tolerance of elderly cancer patients entered onto phase II clinical trials: an Illinois cancer center study. J Clin Oncol.

[6] Pang HH, Wang XF, Stinchcombe TE (2016). Enrollment trends and disparity among patients with lung cancer in national clinical trials, 1990 to 2012. J Clin Oncol.

[7] Freedman RA, Foster JC, Seisler DK (2017). Accrual of older patients with breast cancer to alliance systemic therapy trials over time: protocol A151527. J Clin Oncol.

[8] Stinchcombe TE, Zhang Y, Vokes EE (2017). Pooled analysis of individual patient data on concurrent chemoradiotherapy for stage III non-small-cell lung cancer in elderly patients compared with younger patients who participated in US National Cancer Institute cooperative group studies. J Clin Oncol.

[9] Ludmir EB, Mainwaring W, Lin TA (2019). Factors associated with age disparities among cancer clinical trial participants. JAMA Oncol.

[10] Ehrhardt S, Appel LJ, Meinert CL (2015). Trends in National Institutes of Health Funding for clinical trials registered in ClinicalTrials.gov. JAMA..

[11] Ross JS, Mulvey GK, Hines EM, Nissen SE, Krumholz HM (2009). Trial publication after registration in ClinicalTrials.Gov: a cross-sectional analysis. PLoS Med..

[12] Califf RM, Zarin DA, Kramer JM, Sherman RE, Aberle LH, Tasneem A (2012). Characteristics of clinical trials registered in ClinicalTrials.gov, 2007–2010. JAMA..

[13] Kim HJ, Fay MP, Feuer EJ, Midthune DN (2000). Permutation tests for joinpoint regression with applications to cancer rates. Stat Med.

[14] Zhang ZJ, Schon L (2022). The current status of clinical trials on biologics for cartilage repair and osteoarthritis treatment: an analysis of ClinicalTrials.gov data. Cartilage..

[15] National Institutes of Health, National Cancer Institute, Surveillance Epidemiology, and End Results Cancer Statistics. SEER Cancer Statistics Review 1975–2018: Table 1.11, Median age of cancer patients at diagnosis, 2014–2018 by primary cancer site, race and sex. https://seer.cancer.gov/archive/csr/1975_2018/browse_csr.php?sectionSEL=1&pageSEL=sect_01_table.11. Accessed: 11 June 2023.

[16] Center for Drug Evaluation. Annual Report on the Progress of Clinical Trials for New Drug Registration in China (2021). 2022; Available at: https://www.cde.org.cn/main/news/viewInfoCommon/1839a2c931e1ed43eb4cc7049e189cb0. Accessed 28 June 2023.

[17] Lewis JH, Kilgore ML, Goldman DP (2003). Participation of patients 65 years of age or older in cancer clinical trials. J Clin Oncol.

[18] World Health Organization. Cardiovascular Diseases. Available at: https://www.who.int/health-topics/cardiovascular-diseases. Accessed 28 June 2023.

[19] Bourgeois FT, Orenstein L, Ballakur S, Mandl KD, Ioannidis JPA (2017). Exclusion of elderly people from randomized clinical trials of drugs for ischemic heart disease. J Am Geriatr Soc.

[20] Herrera AP, Snipes SA, King DW, Vigil IT, Goldberg DS, Weinberg AD (2010). Disparate inclusion of older adults in clinical trials: priorities and opportunities for policy and practice change. Am J Public Health..

[21] Shenoy P, Harugeri A (2015). Elderly patients' participation in clinical trials. Perspect Clin Res.

[22] Mangoni AA, Jackson SHD (2004). Age-related changes in pharmacokinetics and pharmacodynamics: basic principles and practical applications. Br J Clin Pharmacol.

[23] Earl JK, Gerrans P, Hunter M (2017). Better ways of assessing cognitive health.

[24] US. Food & Drug Administration. Decentralized clinical trials (DCT) draft guidance. Available at: https://cacmap.fda.gov/drugs/news-events-human-drugs/decentralized-clinical-trials-dct-draft-guidance-06202023. Accessed 28 June 2023.

[25] U.S. Department of Health and Human Services Food and Drug Administration Oncology Center of Excellence. Center for Biologics Evaluation and Research (CBER). Inclusion of older adults in cancer clinical trials guidance for industry. 2022.

